# Screening of ectoparasites from domesticated dogs for bacterial pathogens in Vientiane, Lao PDR

**DOI:** 10.1111/zph.12753

**Published:** 2020-07-10

**Authors:** Hung Manh Nguyen, Watthana Theppannga, Khamsing Vongphayloth, Bounlom Douangngeun, Stuart D. Blacksell, Matthew T. Robinson

**Affiliations:** ^1^ Lao Tropical and Public Health Institute Vientiane Laos; ^2^ National Animal Health Laboratories Vientiane Laos; ^3^ Institut Pasteur du Laos Vientiane Laos; ^4^ Mahidol‐Oxford Tropical Medicine Research Unit Bangkok Thailand; ^5^ Centre for Tropical Medicine, Nuffield Department of Medicine University of Oxford Oxford UK; ^6^ Lao‐Oxford‐Mahosot Hospital‐Wellcome Trust Research Unit (LOMWRU), Microbiology Laboratory Mahosot Hospital Vientiane Laos; ^7^ Present address: Faculty of Medicine Faculty of Nursing Sciences Université Laval Québec QC Canada

**Keywords:** arthropod, domesticated animals, flea, Laos, lice, ticks

## Abstract

Arthropod‐borne diseases are widespread worldwide and are a complex interaction between animals, humans and ectoparasites. The understanding of the diversity and epidemiology of organisms transmitted by arthropod vectors, and the role of hosts and vectors in transmission of infections remain limited in Lao PDR. What knowledge does exist is primarily focused on more rural regions of the country. This study screened ectoparasites from domestic dogs in Vientiane city for the presence of bacterial pathogens of zoonotic importance. A total of 3,511 arthropod vectors were collected from 112 dogs. Vectors collected were *Rhipicephalus sanguineus* ticks, *Ctenocephalides felis felis and Ctenocephalides felis orientis* fleas and *Heterodoxus spiniger* lice. A sub‐sample of vectors from each dog was analysed by PCR to identify the potential bacteria. From 129 vector pools, *Rickettsia* spp. was detected in 6.7% (7/105) pools of ticks, 86.4% (19/22) pools of fleas and both pools of lice. Sequencing analysis confirmed *Rickettsia felis* in 13 flea pools and one louse pool and *Rickettsia asembonensis* in six flea pools. Anaplasmataceae was identified in 14.3% (15/105) tick pools and 100% (22/22) flea pools. Sequencing revealed the presence of *Anaplasma platys* in ticks and *Wolbachia pipientis* in fleas. *Leptospira* spp. was detected in one tick and one louse pool, and *Brucella* spp. was detected in 12.4% (13/105) tick pools. All samples were negative for *Bartonella* spp., *Coxiella burnetii* and *Borrelia burgdorferi*. This is the first study providing evidence of *R. asembonensis* in fleas in Laos. Results from this study show arthropods are potential vectors to transmit zoonotic infection in Vientiane city, suggesting humans are at risk of zoonotic infections in the city.

**Impacts**

- Domestic dogs in Lao PDR harbour a large number of arthropods including ticks, fleas and lice, which are known to be able to transmit bacterial pathogens of human and veterinary importance.
- A number of bacterial species were identified within these vectors, many of which are known to be able to cause disease in humans and animals.
- Due to the high number of domestic and feral dogs in Vientiane, and the large number of vectors, there is a high risk of transmission of these pathogens between dogs and humans within the city.

## 1 INTRODUCTION

The requirement of haematophagy and the ability to feed off different hosts (either in one life stage or across multiple life stages) mean ectoparasites are highly efficient transmitters of zoonotic diseases and, as such, are known vectors for a number of bacterial and viral pathogens of both veterinary and clinical importance (Leitner, Wali, Kincaid, & Costero‐Saint Denis, 2015). Excellent examples are fleas and ticks, which are able to transmit a number of bacteria, many of which have been identified as emerging vector‐borne diseases (Colwell, Dantas‐Torres, & Otranto, 2011). There has been a rise in prevalence of tick‐borne diseases in both clinical and veterinary settings in recent decades. The incidences have been attributed to Ixodidae ticks (hard ticks) as mode of transmission between animals and humans (Vannier & Krause, 2012). Spotted fever group rickettsia (SFGR) are commonly documented in humans who have had contact with animals and those who have reported exposure to arthropods. For instance, *Rickettsia felis* is the most widespread zoonotic pathogen transmitted by fleas (Assarasakorn et al., 2012; Kernif et al., 2012; Troyo et al., 2016; Varagnol et al., 2009), yet the number of actual cases is suspected to be much greater due to non‐reporting and misdiagnosis.

Screening of ectoparasites and vectors in Southeast Asia has identified a number of important pathogens. *Ctenocephalides felis* fleas from cats in Bangkok, Thailand, were found to harbour a variety of *Bartonella* species including *B. henselae*, *B. clarridgeiae* and *B. koehlerae*, whilst in north‐eastern Thailand, strains similar to *B. elizabethae, B. rattimassiliensis, B. rochalimae* and *B. tribocorum* were also reported (Assarasakorn et al., 2012; Billeter et al., 2013). There were various agents identified in ticks from the Thai‐Myanmar border and Vietnam which include *Anaplasma* spp., *Ehrlichia* spp. and *Rickettsia* spp. (Parola et al., 2003).

In Lao People's Democratic Republic (Lao PDR), ticks from Khammouan Province carry *Rickettsia japonica* and *Rickettsia tamurae*, two rickettsia species of known clinical importance, as well as *Borrelia* spp., *Anaplasma* spp. and *Ehrlichia* spp. (Taylor et al., 2016). All these genera contain species of zoonotic relevance. *Rickettsia typhi* was successfully identified in fleas from cats and dogs in Phou Khao Khouay National Park, approximately 100 km from Vientiane (Varagnol et al., 2009), whilst *Rickettsia* spp., *R. felis* and *B. clarridgeiae* have been identified in fleas from dogs in northern Laos (Kernif et al., 2012). As yet no extensive survey has been carried out in Vientiane, the capital of Lao PDR which is a very different environment from the rest of the country. Compared to elsewhere, Vientiane is a highly urbanized environment and is the economic hub of the country, with a population of 700,000 to 1,000,000 people. Population density is high compared to Laos as a whole (6,000/km^2^ versus 130/km^2^). The higher population density, along with pet ownership and a large population of stray dogs and cats, makes the potential for vector transmission of zoonotic pathogens an important aspect of public health.

Considering these aspects, the following study screened ectoparasite vectors from domestic dogs in Vientiane for the presence of bacterial pathogens of zoonotic importance.

## 2 METHODS

Arthropods, including ticks, fleas and lice, were collected from dogs brought to eight veterinary clinics in Vientiane capital. Basic information was collected for each dog, including reason for visit and overall health. Arthropods were morphologically identified (Hopkins & Rothschild, 1953; Price & Graham, 1997; Walker, Keirans, & Horak, 2000; Yamaguti, Tipton, Keegan, & Toshioka, 1971) and pooled according to species, life stage, sex (adult ticks only) and the individual animal they were retrieved from.

DNA from arthropod pools was extracted using DNeasy Blood & Tissue Kit (Qiagen) following the manufacturer's methodology with the following modification: incubation with proteinase K was a minimum of 1 hr to overnight, and DNA was eluted in two 50 μl volumes of TE buffer. DNA samples were assayed by conventional PCR targeting Anaplasmataceae 16S rRNA gene (Parola et al., 2000), and qPCR assays targeting *Rickettsia* spp. 17‐kDa gene (Jiang et al., 2004), *Leptospira* spp. *rrs* gene (Smythe et al., 2002), *Borrelia burgdorferi* 23S rRNA gene (Courtney, Kostelnik, Zeidner, & Massung, 2004), *Bartonella* spp. 23S rRNA gene (BartAF GTG YTT TAT TCT GGT GTT GCT TC, BartAR GCA ATA GCA GCT TCA GCM G, BartAfam 6‐FAM‐TGC WGA TGT TCG YTC TGT TAT GCA TGA AAT GG‐BHQ1, pers. comm. Dr K. Mullins), *Coxiella burnetii* IS1111 gene (Tilburg, Horrevorts, Peeters, Klaassen, & Rossen, 2011) and *Brucella* spp. *bcsp31* gene (Bounaadja et al., 2009). *Rickettsia* spp.‐positive samples were checked for *R. typhi* by qPCR targeting the *ompB* gene (Henry et al., 2007), whilst Anaplasmataceae‐positive samples were checked with qPCRs for *Anaplasma phagocytophilum msp2* gene (Courtney et al., 2004) and *Ehrlichia chaffeensis* 16S rRNA gene (Loftis, Massung, & Levin, 2003).

PCR products from samples positive for *Rickettsia* spp. and Anaplasmataceae were sequenced by Macrogen, and consensus sequences analysed on CLC Main Workbench version 7.8.1 (QIAGEN Aarhus A/S) and BLAST to compare with other sequences available in the GenBank database (http://blast.ncbi.nlm.nih.gov).

## 3 RESULTS

In total, 3,511 arthropods were collected from 112 dogs in Vientiane city. Ticks (*n* = 3,151) were collected from 105 dogs, and all were identified as *Rhipicephalus sanguineus* (brown dog tick). Adults accounted for 74.5% (43.0% of adults were female), whilst nymphs and larvae accounted for 24.8% and 0.7%, respectively. On average, dogs had 19 ticks removed (IQR: 6–45) with one dog having a maximum of 200. Fleas (*n* = 225) were found on 22 dogs and were identified as *Ctenocephalides felis felis* and *Ct. felis orientis*, and the median number of fleas per dog was 4 (IQR: 2–16). Two dogs had lice (*n* = 135), identified as *Heterodoxus spiniger*. There were 15 dogs carrying both ticks and fleas, whilst one dog carrying ticks and lice, and one dog with all three of these vectors. Due to large number of arthropods collected, a sub‐sample from each dog was used for PCR analysis. Adult female ticks were split into 105 tick pools of one to 10 ticks, fleas were split into 22 pools (1 to 20 per pool) and two louse pools (consisting of one and 50 lice).

*Rickettsia* spp. were identified in 21.7% (28/129) of pools including 6.7% (7/105) tick pools, 86.4% (19/22) flea pools and both louse pools (100%, 2/2). No evidence of *R. typhi* was found. Sequencing of the 17‐kDa gene identified two species of *Rickettsia* in flea and louse pools. Fourteen pools showed 99%–100% identity to *R. felis* URRWXCal2 (accession no. CP000053), whilst six pools showed 99%–100% identity to *Rickettsia asembonensis* 8294D3 (MK923744). Eight *Rickettsia* spp.‐positive samples could not be identified to species level (one louse and seven tick pools). *Leptospira* spp. were detected in one tick and one louse pool, and one additional tick pool was likely positive. Anaplasmataceae was identified in 28.7% (37/129) pools (15 tick and 22 flea pools). No pools were positive for *A. phagocytophilum* or *E. chaffeensis*. Two Anaplasmataceae‐positive tick pools showed 100% identity to *Anaplasma platys* (CP046391), whilst one flea pool showed 99% identity to *Wolbachia pipientis* (LN864488). There were 12.4% (13/105) tick pools that were potentially positive with *Brucella* spp. although this could not be confirmed by sequence analysis. All pools were negative for *Bartonella* spp. and *Co. burnetii*, whilst tick pools were negative for *Bo. burgdorferi* (summarized in Table 1). All sequence data were submitted to GenBank database under accession numbers MT469956–MT469975 and MT471981–MT471983.

**TABLE 1:** Summary of pathogens detected. Bracketed values depict number of positive pools in total number of pools tested

Vector species	*Rhipicephalus sanguineus*	*Ctenocephalides felis*	*Heterodoxus spriniger*	Method of identification
*Rickettsia* spp.	6.7% (7/105)	86.4% (19/22)	100% (2/2)	qPCR
*R. felis*		59.1% (13/22)	50% (1/2)	Sequencing of 17kDa gene
*R. asembonensis*		27.3% (6/22)		
Anaplasmataceae	14.3% (15/105)	100% (22/22)		cPCR
*Anaplasma platys*	100% (2/2)^a^			Sequencing of 16s rRNA
*Wolbachia pipientis*		100% (1/1)^a^		
*Leptospira* spp.	1.9% (2/105)		50% (1/2)	qPCR
*Brucella* spp.	12.4% (13/105)			qPCR

^a^ Subset of Anaplasmataceae PCR‐positives were sequenced.

Of 109 dogs, 55.0% were classified as being healthy and were attending the veterinary surgery for routine vaccinations, pregnancy health check, grooming or overnight boarding. Of these, 22 (36.7%) had vectors positive for one or more pathogen (Table 2). Of particular note regarding dogs that were not classified as healthy, both dogs with jaundice had vectors positive for Anaplasmataceae; the two dogs with visible rash were positive for *Rickettsia* spp., *R. felis* and/or Anaplasmataceae; the three dogs with an ‘infection’ were positive for *R. felis* and *R. asembonensis*; and the six dogs classified as being generally unwell had vectors positive for *R. felis*, *R. asembonensis*, Anaplasmataceae and/or *Brucella* spp.

**TABLE 2:** Detection of pathogens in vectors based on health status of the host dog

Health status (*n* = 109)	Number of dogs with positive vectors						
	Positive for one or more bacteria	*Rickettsia* spp.	*Rickettsia felis*	*Rickettsia asembonensis*	Anaplasmataceae	*Brucella* spp.	*Leptospira* spp.
Healthy^a^	22/60 (36.7%)	3 (5.0%)	2 (3.3%)	2 (3.3%)	9 (15.0%)	9 (15.0%)	1 (1.7%)
General unwellness^b^	6/16 (37.5%)		3 (18.8%)	1 (6.3%)	1 (6.3%)	2 (12.5%)	
Abdominal pain/abscess	2/12 (16.7%)			2 (16.7%)	1 (8.3%)	1 (8.3%)	
Infection	3/3 (100%)	2 (66.7%)	1 (33.3%)				
Jaundice	2/2 (100%)				2 (100%)		
Rash	2/2 (100%)	1 (50%)	1 (50%)		1 (50%)		
Still birth	1/1 (100%)		1 (100%)				
Other^c^	8/13 (61.5%)	3 (23.1%)	3 (23.1%)		1 (7.7%)	1 (7.7%)	1 (7.7%)

^a^ ‘Healthy’ includes dogs brought in for grooming, boarding, routine vaccinations or who were pregnant, and who showed no signs of abnormal health.

^b^ ‘General unwellness’ includes dogs appearing ‘sad,’ pale, coughing, fever and/or loss of appetite.

^c^ ‘Other’ health status includes dogs who visited the veterinary surgeons for surgery, were injured or have been diagnosed with tumours, paraplegia or canine distemper virus (CDV).

## 4 DISCUSSION

Although data on the presence of zoonotic pathogens in arthropod vectors in Lao PDR exists, much of this is related to rural areas (Kernif et al., 2012; Taylor et al., 2016; Varagnol et al., 2009), there is very little information on the presence of zoonotic arthropod‐borne diseases in the urban setting of Vientiane, outside of dengue. With the high proportion of companion animals (in particular dogs and cats) interacting with large feral populations, there could conceivably be increased risk of zoonotic pathogens transmitted between animals, including pets, and humans. The aim of this survey was to obtain a better understanding of prevalence and diversity of vectors and pathogens from domestic dogs in the capital of Lao PDR. Only three species of vectors were identified: the tick *Rh. sanguineus*, the flea *Ct. felis* (two subspecies identified) and the louse *H. spiniger*. The brown dog tick, *Rh. sanguineus*, is the most widespread tick in the world (Dantas‐Torres, 2008) and a known vector for transmission for both canine and human diseases (Shaw, Day, Birtles, & Breitschwerdt, 2001). It is interesting that only *Rh. sanguineus* was identified, despite other studies identifying a greater diversity of species, although the majority of studies were in rural areas of Laos (Taylor et al., 2016; Vongphayloth, Brey, Robbins, & Sutherland, 2016). This observation could be explained by the fact that this species has outstanding adaptive capacity to human dwellings due to its close evolutionary relationship with the domestic dog (Gray, Dantas‐Torres, Estrada‐Pena, & Levin, 2013), and in this study, only dog ticks in urban areas were collected. Only *Ct. felis*, with two subspecies *Ct. felis felis* and *Ct. felis orientis*, were detected here. Whilst *Ct. felis* has been identified in previous studies, *Pulex irritans* and *Xenopsylla cheopis* have also been collected from dogs in northern Laos (Kernif et al., 2012). *Heterodoxus spiniger* has been previously identified in Laos (Beaucournu, Jouan, & Menier, 2001).

*Rickettsia felis* and *R. asembonensis* were detected in fleas and lice. In Laos, *R. felis* has been found in fleas collected from domesticated dogs outside of Vientiane (Varagnol et al., 2009) and has been confirmed as a human pathogen in Laos (Dittrich et al., 2014; Mayxay et al., 2015; Phongmany et al., 2006) suggesting potential interactions between humans and vectors. *Rickettsia asembonensis* has been mapped worldwide (Maina et al., 2019) and is confirmed in Southeast Asia, including Thailand and Malaysia (Low et al., 2017; Odhiambo, Maina, Taylor, Jiang, & Richards, 2014). This study reports the first detection, to our knowledge, of *R. asembonensis* in Laos. Although *R. asembonensis* is closely related to *R. felis*, little is known of its pathogenicity to humans although there is some molecular evidence (*gltA* and *ompB* sequences) of infection in humans and monkeys in Malaysia (Tay, Koh, Kho, & Sitam, 2015). Similar to many reports that fleas are the most common vector to be infected with *R. asembonensis*, this study only identified this agent in *Ct. felis* (Kocher et al., 2016; Maina et al., 2016; Oteo et al., 2014; Silva et al., 2017; Troyo et al., 2016). Other arthropods have been found to be infected with *R. asembonensis* including *Pulex simulans* and *Amblyomma ovale* from dogs and *Rhipicephalus microplus* from cows (Troyo et al., 2016); yet, ticks and lice were negative for *R. asembonensis* in our sample; and further investigation is needed to confirm the distribution of this agent in the country.

This study presented evidence of *Leptospira* spp. in *Rh. sanguineus* and *H. spiniger*. Canine leptospirosis has been demonstrated in a number of studies, suggesting a risk of human infection (Gay, Soupe‐Gilbert, & Goarant, 2014; Weekes, Everard, & Levett, 1997). There are also questions as to whether *Leptospira* spp. can be vector transmitted (Wojcik‐Fatla et al., 2012) although it is likely that ticks and lice here fed off an infected dog and it is the contaminated blood meal that is being detected.

*Anaplasma platys* and *W. pipientis* were detected in tick and flea pools, respectively. *Anaplasma platys* infection has been commonly reported in dogs and is described as being a potential human pathogen (Arraga‐Alvarado et al., 2014; Geurden et al., 2018; Maggi, Mascarelli, Havenga, Naidoo, & Breitschwerdt, 2013; Matei et al., 2016). *Rhipicephalus sanguineus* ticks have been identified as the dominant vector in the transmission of *A. platys* (Cicuttin et al., 2014; Geurden et al., 2018; Ramos et al., 2014; Silva et al., 2016). *Wolbachia* spp. are common endosymbionts found in a number of insects (Gorham, Fang, & Durden, 2003); however, we are unaware of any evidence of human infestation.

A number of tick pools were suspected to be *Brucella* spp. positive, although this could not be confirmed by sequence analysis. Transmission of *Brucella* spp. is via contact exposure to infected animals or by environmental contamination with infected fluids, although a number of studies have suggested the possibility of arthropod‐borne transmission (Kosoy & Goodrich, 2018). As with *Leptospira* spp., it is likely that ticks fed on an infected dog and again we are detecting the blood meal. Either way, the possibility of *Brucella* spp. in pet dogs raises important public health implications. Previous work has serologically confirmed *Brucella* spp. in goats in Vientiane capital (Burns et al., 2018), and therefore, more work is needed to confirm the presence of *Brucella* spp. in dogs and *Rh. sanguineus* ticks.

Interestingly, the majority of dogs were classified as being healthy and showed no signs of an infection. Previously, it has been shown that the majority of dogs infected with *A. platys* show no clinical signs (Arraga‐Alvarado et al., 2014; Shaw et al., 2001), and therefore, it is not surprising to obtain positive results from healthy dogs. If symptoms are present, both *Rickettsia* and *Anaplasma* usually have unspecific and mild presentations; both *Rickettsia* spp. and *A. platys* are known to cause anorexia, depression, fever and pale mucous membranes, all three of which were reported in 12 of these dogs.

## 5 CONCLUSION

This study highlights the prevalence of vectors and associated pathogens harboured on domesticated dogs in Vientiane, Lao PDR. Evidence on rickettsial infection with the presence of *R. felis* and the first detection of *R. asembonensis* in arthropods in the country shows a high risk of infection to humans. Many other pathogens were also identified emphasizing that arthropods are potential vectors to transmit zoonotic infection. With both *Rh. sanguineus* and *C. felis* known to cause infestations within households (Hansford, Pietzsch, Cull, & Medlock, 2010; Rust & Dryden, 1997), control of arthropod vectors on domestic animals is both a veterinary and public health importance in the city of Vientiane, requiring greater attention in clinical diagnosis, treatment and prevention strategies.

## CONFLICT OF INTEREST

The authors declare no conflict of interest.
